# Effects of electrical muscle stimulation on cerebral blood flow

**DOI:** 10.1186/s12868-021-00670-z

**Published:** 2021-11-14

**Authors:** Soichi Ando, Yoko Takagi, Hikaru Watanabe, Kodai Mochizuki, Mizuki Sudo, Mami Fujibayashi, Shinobu Tsurugano, Kohei Sato

**Affiliations:** 1 Graduate School of Informatics and Engineering, The University of Electro-Communications, 1-5-1 Chofugaoka, Chofu, Tokyo, 182-8585 Japan; 2 Faculty of Informatics and Engineering, The University of Electro-Communications, 1-5-1 Chofugaoka, Chofu, Tokyo, 182-8585 Japan; 3 Physical Fitness Research Institute, Meiji Yasuda Life Foundation of Health and Welfare, Tobuki 150, Hachioji, Tokyo, 192-0001 Japan; 4 Faculty of Agriculture, Setsunan University, Osaka, Japan; 5 Health Care Center, The University of Electro-Communication, 1-5-1 Chofugaoka, Chofu, Tokyo, 182-8585 Japan; 6 Department of Arts and Sport Science, Tokyo Gakugei University, Tokyo, Japan

**Keywords:** Brain, Cerebral perfusion, Skeletal muscle, Neuromuscular stimulation, CO_2_, Neural activation

## Abstract

**Background:**

Electrical muscle stimulation (EMS) induces involuntary muscle contraction. Several studies have suggested that EMS has the potential to be an alternative method of voluntary exercise; however, its effects on cerebral blood flow (CBF) when applied to large lower limb muscles are poorly understood. Thus, the purpose of this study was to examine the effects of EMS on CBF, focusing on whether the effects differ between the internal carotid (ICA) and vertebral (VA) arteries.

**Methods:**

The participants performed the experiments under EMS and control (rest) conditions in a randomized crossover design. The ICA and VA blood flow were measured before and during EMS or control. Heart rate, blood pressure, minute ventilation, oxygen uptake, and end-tidal partial pressure of carbon dioxide (P_ET_CO_2_) were monitored and measured as well.

**Results:**

The ICA blood flow increased during EMS [Pre: 330 ± 69 mL min^−1^; EMS: 371 ± 81 mL min^−1^, P = 0.001, effect size (Cohen’s d) = 0.55]. In contrast, the VA blood flow did not change during EMS (Pre: 125 ± 47 mL min^−1^; EMS: 130 ± 45 mL min^−1^, P = 0.26, effect size = 0.12). In the EMS condition, there was a significant positive linear correlation between ΔP_ET_CO_2_ and ΔICA blood flow (R = 0.74, P = 0.02). No relationships were observed between ΔP_ET_CO_2_ and ΔVA blood flow (linear: R = − 0.17, P = 0.66; quadratic: R = 0.43, P = 0.55).

**Conclusions:**

The present results indicate that EMS increased ICA blood flow but not VA blood flow, suggesting that the effects of EMS on cerebral perfusion differ between anterior and posterior cerebral circulation, primarily due to the differences in cerebrovascular response to CO_2_.

## Background

Sedentary behavior and physical inactivity are associated with numerous negative health concerns [1]. Several studies suggest that electrical muscle stimulation (EMS) applied to the large lower limb muscles can be used as an alternative modality to voluntary exercise. For example, EMS of large muscles increases whole-body glucose uptake [2] and lowers postprandial hyperglycemia in patients with type 2 diabetes [3]. Furthermore, EMS training improves muscle strength [4, 5] and prevents muscle atrophy after surgery [6] or during hospitalization [7]. Thus, EMS may be potentially beneficial for individuals who are unable to exercise as well as healthy populations.

In contrast to the beneficial effects of EMS on muscle strength and metabolism, its effects on cerebral perfusion are poorly understood. It is well established that acute voluntary exercise increases cerebral blood flow (CBF) [8]. Voluntary exercise induces many physiological changes that originate centrally (brain activity associated with central motor command), peripherally (muscle contraction and resultant physiological changes), and under cardiovascular command [9]. Conversely, EMS induces involuntary muscle contraction without a central motor or cardiovascular command, which enables isolation of the physiological changes derived from muscle contraction. Hence, measuring CBF during EMS would identify the contribution of muscle contraction and the resultant physiological changes to CBF regulation. Furthermore, sedentary aging is associated with a decline in CBF [10, 11], and this decline seems to be linked to cognitive impairments [12, 13]. If EMS increases CBF similar to voluntary exercise [8], EMS may be implicated as a therapeutic strategy to maintain brain health, particularly for those who are unable to exercise.

Blood supply to the brain originates from the internal carotid artery (ICA) and vertebral artery (VA). The ICA supplies blood to large parts of the cerebral cortex, while the VA supplies blood to the brain stem, cerebellum, and spinal cord [14]. The cerebrovascular response to arterial carbon dioxide (CO_2_), which is termed cerebrovascular CO_2_ reactivity, is higher in the anterior cerebral circulation, supplied by the ICA, than in the posterior cerebral circulation, supplied by the VA [15]. These results suggest that CBF responses to physiological stress are different between ICA and VA blood flow, and lower CO_2_ reactivity in the posterior circulation may serve to preserve blood flow and maintain vital systemic functions [15]. Hence, it is expected that measuring both ICA and VA blood flow would characterize cerebral perfusion in response to physiological changes induced by EMS.

Given this background, this study aimed to examine the differential effects of EMS on CBF, and whether the effects of EMS on cerebral perfusion differed between anterior and posterior cerebral circulation. The current findings may provide insight into the potential effects of EMS on brain health as an alternative exercise modality.

## Methods

### Participants

Ten healthy male participants were recruited for this study. However, CBF data from one participant were removed because of technical issues. Data from nine healthy male participants (Age: 22.7 ± 1.6 yr., height: 173.3 ± 4.8 cm, mass: 71.5 ± 8.4 kg) were then analyzed. The participants had no history of cerebrovascular, cardiovascular, or respiratory diseases and were not taking any medications. They were asked to refrain from intense physical activity for 24 h and not consume any food or drink, except water, 3 h before the main experiments. The study was approved by the University of Electro-Communications Human Ethics Committee (18008). The study also conformed to the standards set by the latest revision of the Declaration of Helsinki, except for registration in a database, and each participant provided written informed consent.

### Experimental procedure

**Fig. 1:**
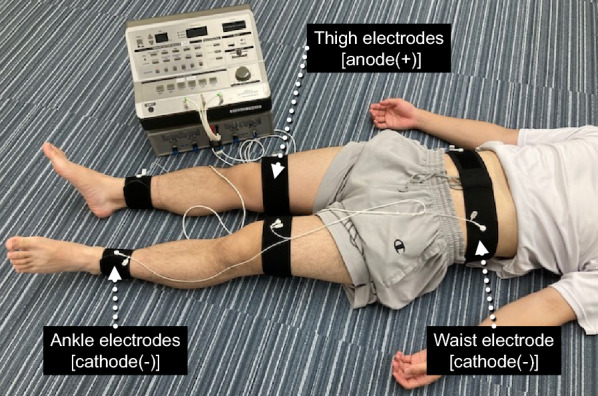
A picture of electrical muscle stimulation (EMS). Belt electrodes (anode and cathode) were attached to the waist and bilateral distal parts of the thigh and ankle with straps. EMS was applied to the abdomen, glutes, thighs, and lower legs

The participants visited the laboratory on three separate occasions. At the initial visit, the EMS intensity was adjusted for each participant. Belt electrodes were attached to the waist and bilateral distal parts of the thigh and ankle with straps (Fig. 1). EMS was applied to the abdomen, gluteal, thigh, and leg muscle groups using an electrical simulator (Auto Tens Pro; Homer ion, Tokyo, Japan) while lying supine on a bed. The stimulator current waveform was set at a frequency of 4 Hz with a pulse width of 0.25 ms [16]. The current waveform was designed to exponentially increase the pulse, which reduced discomfort during EMS [6]. The stimulus intensity was gradually increased and was set to the maximal tolerable level for each participant [2]. In the present study, the peak stimulus intensities were 120 ± 48 mA (left thigh), 125 ± 59 mA (right thigh), 82 ± 25 mA (left lower leg), and 88 ± 31 mA (right lower leg).

On the second and third visits, the participants performed two experimental conditions: EMS and control. Both experimental conditions were performed in a randomized crossover design and were separated by a minimum of 3 days. In the EMS condition, EMS was applied at a pre-determined intensity for 20 min in the supine position. In the control condition, the participants remained supine for 20 min without EMS. The ambient temperature was maintained at 22–23 °C throughout the experiment.

### Measurement

All variables were measured before and during the EMS or control. Heart rate (HR) was recorded using an HR monitor (V800; Polar Electro Oy, Kempele, Finland). Systolic and diastolic blood pressures were measured in the left arm (HEM-7281 T; Omron, Kyoto, Japan). Mean arterial pressure (MAP) was calculated as [(2 × diastolic blood pressure) + systolic blood pressure]/3. Minute ventilation (V̇_E_), oxygen uptake (V̇O_2_), and end-tidal partial pressure of CO_2_ (P_ET_CO_2_) were sampled through a leak-free mask and measured using a gas analysis system (AE-300; Minato Medical Science, Tokyo, Japan).

The right ICA and VA blood flow were measured using a color-coded ultrasound system (Vivid-i GE Healthcare, Tokyo, Japan). Details of the measurements are described elsewhere [17]. In brief, the ICA blood flow was measured ~ 1.0–1.5 cm above the carotid bifurcation. VA blood flow was measured between the transverse processes of C3 and the subclavian artery. During the Doppler measurement, the operator ensured that the insonation angle did not vary from 60°. Blood flow was calculated using the brightness mode-determined diameter and pulse-wave Doppler mode-determined blood velocity. Blood flow velocity measurements were averaged across ~ 15 cardiac cycles to account for the oscillatory effects caused by respiration. Cerebrovascular conductance (CVC) was calculated from the ratio of ICA and VA blood flow to the MAP. Global CBF (gCBF) was calculated as the sum of the blood flow in the ICA and VA [(ICA blood flow + VA blood flow) × 2 (mL min^−1^)].

### Data and statistical analysis

HR, V̇_E_, V̇O_2_, and P_ET_CO_2_ were averaged over 1 min before and during the last 1 min of EMS or control. The normal distribution of data was confirmed using the Shapiro–Wilk test. All data were analyzed using a two-way repeated-measures analysis of variance [condition (EMS and control) × time (pre- and during EMS/rest)]. A post-hoc analysis was performed using paired sample t-tests with Bonferroni correction. For the ICA and VA blood flow in the EMS condition, effect size was calculated using Cohen’s d with a small effect designated as 0.2, medium as 0.5, and large as 0.8 [18]. In the EMS condition, Pearson’s correlation test was used to establish a correlation between changes in P_ET_CO_2_ (ΔP_ET_CO_2_) and ICA (ΔICA) blood flow and ΔP_ET_CO_2_ and changes in VA (ΔVA) blood flow. All statistical analyses were performed using SPSS (version 25.0; SPSS Inc., Chicago, IL, USA). Data are expressed as mean ± SD. Statistical significance was set at P < 0.05.

## Results

Table 1 summarizes cardiorespiratory and cerebrovascular variables. Significant increases were observed in HR (P = 0.01), MAP (P = 0.005), V̇_E_ (P = 0.001), and V̇O_2_ (P = 0.001) during EMS. There was a significant main effect of time on P_ET_CO_2_ (P = 0.048), which indicates that P_ET_CO_2_ increased during the experiment. The ICA blood flow increased during EMS (P = 0.001, effect size = 0.55), while the VA blood flow did not change during EMS (P = 0.26, effect size = 0.12). As a result, gCBF increased during EMS (P < 0.001). The ICA diameter (P = 0.02) and ICA CVC (P = 0.02) increased during EMS. In the control condition, ICA blood flow decreased (P = 0.02). However, other cardiorespiratory and cerebrovascular variables did not change in the control condition.

**Table 1:** Cardiorespiratory and cerebrovascular variables before and during electrical muscle stimulation (EMS) or rest (control)

Variable	EMS		Control		P value		
					Main effect		Interaction
	Pre	EMS	Pre	Rest	Condition	Time	
HR (bpm)	63± 8	84 ± 17*	62 ± 10	61 ± 9	P = 0.01	P = 0.03	P = 0.005
MAP (mmHg)	87 ± 4	95 ± 6**	86 ± 3	85 ± 4	P < 0.001	P = 0.03	P = 0.001
V̇_E_, l·min^-1^	9.9 ± 2.1	20.7 ± 6.7^**^	9.0 ± 1.8	7.9 ± 0.9	P = 0.001	P = 0.004	P = 0.001
V̇O_2_, mL min^-1^	255 ± 33	696 ± 268**	263 ± 39	240 ± 23	P = 0.001	P = 0.001	P = 0.001
P_ET_CO_2_, mmHg	41.5 ± 2.7	43.1 ± 2.3	42.0 ± 3.3	42.1 ± 2.9	P = 0.67	P = 0.048	P = 0.25
ICA blood flow (ml min^-1^)	330 ± 69	371 ± 81**	346 ± 88	329 ± 78 *	P = 0.18	P = 0.04	P = 0.001
Diameter (mm)	0.48 ± 0.04	0.50 ± 0.04 *	0.48 ± 0.05	0.48 ± 0.04	P = 0.25	P = 0.08	P = 0.049
Blood flow velocity (cm sec^-1^)	30.3 ± 2.7	31.6 ± 3.2	31.6 ± 4.0	29.9 ± 2.5	P = 0.85	P = 0.76	P = 0.04
ICA CVC (ml·min^-1^ mmHg^-1^)	3.79 ± 0.80	3.93 ± 0.86*	4.01± 1.00	3.85 ± 0.87	P = 0.50	P = 0.83	P = 0.005
VA blood flow (ml min^-1^)	125 ± 47	130 ± 45	122 ± 41	122± 41	P = 0.58	P = 0.50	P = 0.16
Diameter (cm)	0.33 ± 0.05	0.32 ± 0.05	0.32 ± 0.04	0.32 ± 0.05	P = 0.61	P = 0.86	P = 0.49
Blood flow velocity (cm sec^-1^)	24.1 ± 4.1	25.5 ± 3.7	24.2 ± 3.3	24.2 ± 3.5	P = 0.67	P = 0.27	P = 0.17
VA CVC (ml·min^-1^ mmHg^-1^)	1.42 ± 0.51	1.36 ± 0.44	1.41 ± 0.46	1.43 ± 0.49	P = 0.77	P = 0.77	P = 0.09
gCBF blood flow (ml min^-1^)	910 ± 192	1,002 ± 199***	937 ± 203	902 ± 184	P = 0.04	P = 0.08	P < 0.001

Values are mean±SD

*HR* heart rate, *MAP* mean arterial pressure, *V̇*_*E*_ minute ventilation, *V̇O*_*2*_ oxygen uptake, *P*_*ET*_*CO*_*2*_ end-tidal partial pressure of CO_2_, *ICA* internal carotid artery, *CVC* cerebrovascular conductance, *VA* vertebral artery, *gCBF* global cerebral blood flow

***p < 0.001, **p < 0.01, *p < 0.05 vs. pre

**Fig. 2:**
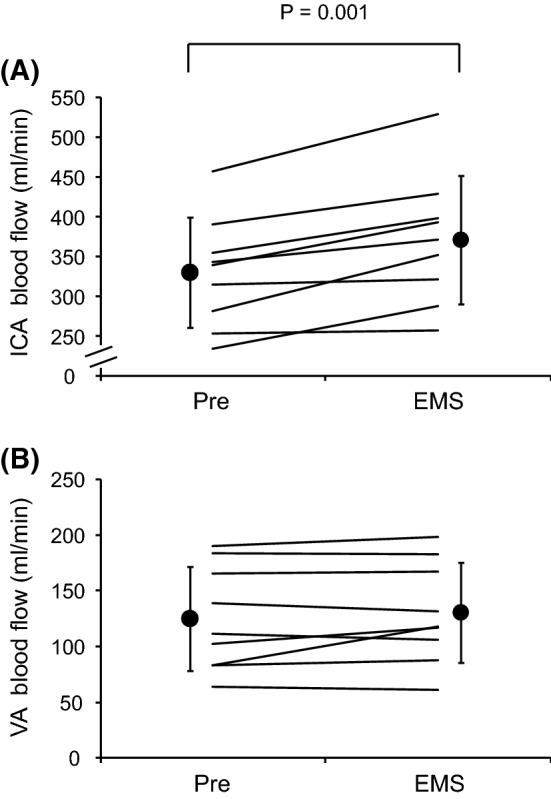
The internal carotid artery (ICA) (**A**) and vertebral artery (VA) (**B**) blood flow in the EMS condition. Lines represent individual data (N = 9). Filled circles and bars represent mean ± standard deviation

**Fig. 3:**
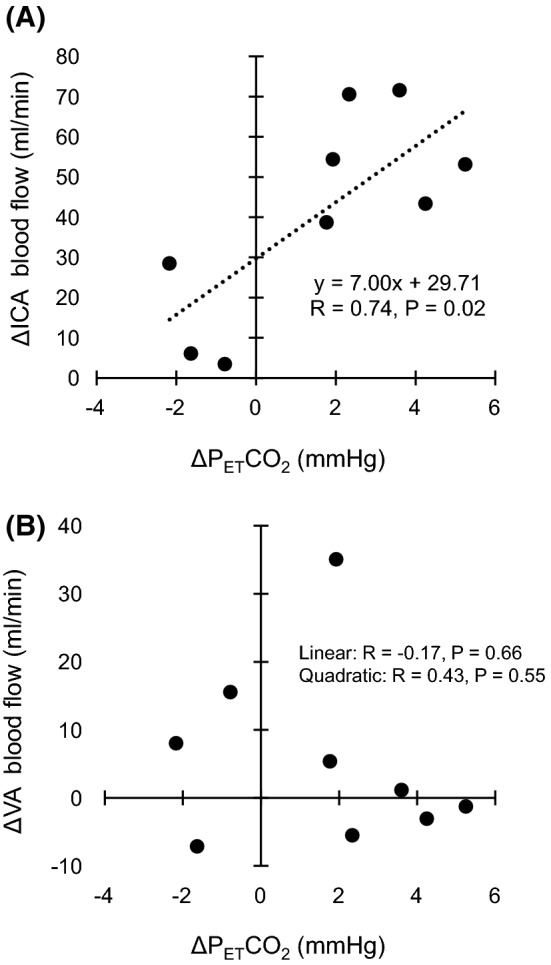
Scatter plots of the relationship between changes in P_ET_CO_2_ (ΔP_ET_CO_2_) and ICA (ΔICA) blood flow (**A**) and between ΔP_ET_CO_2_ and changes in VA (ΔVA) blood flow (**B**) in the EMS condition

Figure 2 displays individual and mean data of the ICA and VA blood flow in the EMS condition. The ICA blood flow markedly increased for seven of nine participants. Conversely, the VA blood flow remained almost unchanged for seven of nine participants. Figure 3 illustrates scatter plots of the relationship between ΔP_ET_CO_2_ and ΔICA blood flow and between ΔP_ET_CO_2_ and ΔVA blood flow in the EMS condition. A significant positive correlation was observed between ΔP_ET_CO_2_ and ΔICA blood flow (R = 0.74, P = 0.02). In contrast, no relationships were observed between ΔP_ET_CO_2_ and ΔVA blood flow (linear: R = − 0.17, P = 0.66; quadratic: R = 0.43, P = 0.55).

## Discussion

The present study examined how EMS on large muscles influences CBF. The major findings of this study were that (1) EMS increased the ICA blood flow, but not the VA blood flow, and that (2) there was a positive linear correlation between ΔP_ET_CO_2_ and ΔICA blood flow in response to EMS, but not between ΔP_ET_CO_2_ and ΔVA blood flow. These results indicate that EMS affects the anterior and posterior cerebral circulation differently, primarily due to the differences in cerebrovascular response to CO_2_.

CBF is regulated via dynamic cerebral autoregulation over a wide range of cerebral perfusion pressures [8]. During exercise, CBF is regulated by interactions between neural activity and metabolism, blood pressure, sympathetic nervous system activity, partial pressure of arterial O_2_ and CO_2_, and cardiac output [8, 19]. In particular, CBF is highly sensitive to changes in the partial pressure of arterial CO_2_ [8, 20]. The cerebrovascular response to CO_2_ is a vital homeostatic function that helps regulate and maintain central pH, and therefore, affects the central respiratory chemoreceptor stimulus [20]. P_ET_CO_2_ increases during low to moderate voluntary exercise [17, 21], and the increase in P_ET_CO_2_ is ascribed to the increase in CO_2_ production from contracting muscles, pulmonary arterial flow, and alveolar parameters related to breathing [22]. In this study, an increase in P_ET_CO_2_ was observed. EMS induces involuntary muscle contraction. Thus, the increase in P_ET_CO_2_ would be primarily attributable to CO_2_ production from the contracting muscle. Importantly, a significant positive linear correlation was observed between ΔP_ET_CO_2_ and ΔICA blood flow, with an R-squared value of 0.74. This result indicates that the increase in P_ET_CO_2_ explained 55% of the increase in ICA blood flow. Elevated arterial pressure of CO_2_ leads to vasodilation of cerebral arterioles in the downstream bed to wash out CO_2_ from the brain tissue [20]. Given that the ICA supplies a large portion of the brain, these results suggest that the increase in ICA blood flow contributed to the removal of CO_2_ from the brain. Furthermore, an increase in the ICA CVC during EMS suggests that vascular beds were vasodilated in the anterior cerebral circulation. The increase in the ICA CVC appears to corroborate the notion that an increase in P_ET_CO_2_ is linked to an increase in ICA blood flow.

In the present study, the y-intercept of the regression line between ΔP_ET_CO_2_ and ΔICA blood flow was approximately 30 mL min^−1^. This means that ΔP_ET_CO_2_ is not the sole factor responsible for the increase in ICA blood flow during EMS. Rather, there are other physiological factors involved in the increase in ICA blood flow. An increase in MAP during the EMS was observed. Although the influence of exercise-induced increase in MAP on CBF is difficult to discern from other factors [19], the increase in MAP may reasonably be expected to contribute to the observed increase in ICA blood flow. The role of sympathetic nervous activity in CBF regulation is controversial [19, 23], but sympathetic nervous activation might have affected ICA blood flow in the present study. In addition, the HR significantly increased during EMS; hence, changes in MAP, sympathetic nervous system activation, and cardiac output may be at least partly responsible for the increase in ICA blood flow.

Additionally, neural activation is another candidate to account for the increase in ICA blood flow during EMS. The brain receives multiple afferent inputs from contracting muscles. EMS in large muscles induces muscle contraction and increases blood lactate concentration [2, 24]. Thus, the exercise pressor reflex (mechano- and metaboreflex) [25] is thought to be activated during EMS. In the cranium, the ICA is divided into two main branches: the anterior cerebral artery (ACA) and middle cerebral artery (MCA). In the present study, EMS was applied to large muscles, including the abdomen, gluteal, thigh, and leg muscles. The ACA supplies the motor and sensory cortices associated with the cortical representation of the leg [26], while the MCA is the largest terminal branch of the ICA and supplies the motor and sensory cortices associated with the representation of the hip and trunk [27]. Sander et al. indicated that the primary and secondary somatosensory cortices are activated not only during handgrip exercise but also during post-exercise ischemia [28], suggesting that these areas are activated when muscle metaboreflex activation is isolated. Indeed, muscle metaboreflex activation during and after exercise increases ICA blood flow and MCA blood flow velocity [29]. Hence, it is plausible that neural activation in the sensorimotor areas also contributed to the increase in ICA blood flow in the present study.

The results showed that VA blood flow did not change during EMS. There was no correlation between ΔP_ET_CO_2_ and ΔVA blood flow, and VA CVC did not change. VA supplies blood to areas that include the respiratory and cardiovascular control centers, which are critical for vital systemic functions [30]. Hence, the absence of changes in VA blood flow during EMS suggests that posterior circulation is robust to physiological changes induced by muscle contraction, which is advantageous for maintaining homeostatic function.

In the present study, although a moderate effect size was observed of the ICA blood flow increase, the number of participants was small. However, retrospective power analysis indicated that nine participants were adequate to achieve a power of 80% with an alpha of 0.05. This is the first study to evaluate cerebral circulation in response to EMS and can be an important starting point, emphasizing the importance of follow-up clinical trials with larger sample sizes.

This study has some limitations. First, the physiological mechanisms responsible for the increase in ICA blood flow remain to be elucidated. Further studies are also required to understand the physiological mechanisms underlying the differential effects of EMS on the anterior and posterior cerebral circulation. Second, the circle of Willis is known to show considerable anatomical variation [31]. Thus, the possibility that anatomical variation of the circle of Willis influenced the anterior and posterior cerebral circulation in response to EMS cannot be ruled out.

The present study examined a single bout of EMS on cerebral circulation and indicated that EMS increases blood flow to the cerebral cortex. Regular physical activity and cardiorespiratory fitness improvement appear to increase CBF across the lifespan [10, 11]. Hence, a longitudinal study with interventions would be more valuable. Given that EMS is a potential alternative method of exercise, it is worth investigating how long-term EMS training affects cerebral circulation.

## Conclusion

The differential effects of EMS on the anterior and posterior cerebral circulation were examined in this study. While ICA blood flow increased during EMS, VA blood flow did not change, suggesting that the differential responses to EMS between the anterior and posterior cerebral circulations are primarily due to the differences in the cerebrovascular response to CO_2_.

## Data Availability

All data generated or analyzed during this study are included in this published article. Datasets are available from the corresponding author upon request.

## Abbreviations

ACA: Anterior cerebral artery
CBF: Cerebral blood flow
CVC: Cerebrovascular conductance
EMS: Electrical muscle stimulation
gCBF: Global cerebral blood flow
HR: Heart rate
ICA: Internal carotid artery
MAP: Mean arterial pressure
MCA: Middle cerebral artery
P_ET_CO_2_: End-tidal partial pressure of CO_2_
VA: Vertebral artery
V̇E: Minute ventilation
V̇O_2_: Oxygen uptake
